# Predicting delayed methotrexate elimination in pediatric acute lymphoblastic leukemia patients: an innovative web-based machine learning tool developed through a multicenter, retrospective analysis

**DOI:** 10.1186/s12911-023-02248-7

**Published:** 2023-08-03

**Authors:** Chang Jian, Siqi Chen, Zhuangcheng Wang, Yang Zhou, Yang Zhang, Ziyu Li, Jie Jian, Tingting Wang, Tianyu Xiang, Xiao Wang, Yuntao Jia, Huilai Wang, Jun Gong

**Affiliations:** 1 College of Medical Informatics, Chongqing Medical University, Chongqing, China; 2 College of Pharmacy, Chongqing Medical University, Chongqing, China; 3 Big Data Engineering Center, Children’s Hospital of Chongqing Medical University, Chongqing, China; 4 Department of Medicine, Affiliated Hospital of Nantong University, Jiangsu, China; 5 Department of Pharmacy, Children’s Hospital of Chongqing Medical University, Chongqing, China; 6 Department of Information Center, University-Town Hospital of Chongqing Medical University, Chongqing, China

**Keywords:** Methotrexate, Delayed metabolism, Acute lymphoblastic leukemia, Machine learning

## Abstract

**Background:**

High-dose methotrexate (HD-MTX) is a potent chemotherapeutic agent used to treat pediatric acute lymphoblastic leukemia (ALL). HD-MTX is known for cause delayed elimination and drug-related adverse events. Therefore, close monitoring of delayed MTX elimination in ALL patients is essential.

**Objective:**

This study aimed to identify the risk factors associated with delayed MTX elimination and to develop a predictive tool for its occurrence.

**Methods:**

Patients who received MTX chemotherapy during hospitalization were selected for inclusion in our study. Univariate and least absolute shrinkage and selection operator (LASSO) methods were used to screen for relevant features. Then four machine learning (ML) algorithms were used to construct prediction model in different sampling method. Furthermore, the performance of the model was evaluated using several indicators. Finally, the optimal model was deployed on a web page to create a visual prediction tool.

**Results:**

The study included 329 patients with delayed MTX elimination and 1400 patients without delayed MTX elimination who met the inclusion criteria. Univariate and LASSO regression analysis identified eleven predictors, including age, weight, creatinine, uric acid, total bilirubin, albumin, white blood cell count, hemoglobin, prothrombin time, immunological classification, and co-medication with omeprazole. The XGBoost algorithm with SMOTE exhibited AUROC of 0.897, AUPR of 0.729, sensitivity of 0.808, specificity of 0.847, outperforming the other models. And had AUROC of 0.788 in external validation.

**Conclusion:**

The XGBoost algorithm provides superior performance in predicting the delayed elimination of MTX. We have created a prediction tool to assist medical professionals in predicting MTX metabolic delay.

**Supplementary Information:**

The online version contains supplementary material available at 10.1186/s12911-023-02248-7.

## Introduction

Acute lymphoblastic leukemia (ALL) is a prevalent neoplasm in childhood. The incidence of ALL in children below 15 years of age is 0.004%, which accounts for about 35% of all cases of pediatric malignancies [1–3]. Epidemiological studies of ALL indicate that the cumulative incidence is 1/2000 under the age of 15 [4].

Methotrexate (MTX) is a crucial antineoplastic agent in ALL therapy, which inhibits the synthesis of tumor cells by restraining dihydrofolate reductase. In clinical practice, high-dose methotrexate (HD-MTX) can significantly increase the blood drug concentration and permeate blood-brain and blood-testis barriers, so it is recommended as a common chemotherapy approach for ALL treatments. Although HD-MTX is deemed an effective ALL treatment, prolonged exposure to HD-MTX can cause hepatotoxicity, nephrotoxicity, and neurotoxicity [2, 5–8]. A study from China revealed the rate of delayed MTX elimination was as high as 12.1%, which is a non-negligible rate [9]. A clinical trial has demonstrated that 2-12% of patients develop acute kidney injury (AKI) despite appropriate support during HD-MTX treatment [2, 10]. Furthermore, the severity of adverse reactions of MTX is linked to the concentration and duration of drug exposure. Since the liver and immune system of children are not yet fully developed, their tolerance and metabolic capacity to potential liver toxicity of drugs are inadequate. Therefore, children are more prone to delayed MTX elimination, which could affect their prognosis or lead to other adverse outcomes. Consequently, it is crucial to find ways to reduce the delayed elimination of MTX and the incidence of side effects.

To address the problem of delayed MTX elimination, the current approach is to monitor MTX concentration at 24 h, 48 h, and 72 h post-administration and to administer calcium leucovorin rescue agent and urine alkalization if necessary to accelerate MTX elimination. However, the risk of delayed elimination cannot be predicted based on patient’s signs and data before medication. Therefore, early warning and timely intervention are crucial to effectively reduce the risk of delayed MTX elimination and prevent serious adverse drug reactions.

Artificial intelligence (AI) has been widely used in the medical field. In previous studies, machine learning (ML) was used to classify diseases and analyze the survival of prognosis [11, 12]. Researchers not only extracted disease features for building models, but also achieved high accuracy. This can reduce the fluctuation of patient incidence rate and save on medical costs. Therefore, it is necessary to apply ML to predict the metabolic delay of methotrexate. Researchers, such as Wang Yang [13], Yang Fan [14], and Min Zhang [7], have begun using ML to develop prediction models for delayed MTX elimination. However, previous studies have encountered various issues such as small sample sizes, inadequate representation, limited model construction methods, and insufficient comparability. Additionally, predictive indicators failed to fully consider patient clinical data and relevant clinical laboratory indicators.

This study aims to assess the potential correlation between premedication indicators and delayed elimination of MTX by integrating electronic medical data from multiple centers. Furthermore, a prediction model will be developed using ML methods and a web-based tool to offer an early warning for the delayed elimination of MTX in clinical settings.

## Methods

### Study design and population

This retrospective study included MTX dosing information, combination medications and laboratory test indicators from seven affiliated medical institutions of Chongqing Medical University from 2011 to 2017. In addition, for external verification, we used MTX medication data from ALL children in Children’s Hospital affiliated to Chongqing Medical University from 2018 to 2021. Inclusion criteria were: (1) patients ≤ 18 years; (2) ALL with risk classification, morphotyping, and immunological classification; (3) chemotherapy with MTX during hospitalization; (4) MTX blood concentration was measured during hospitalization and not longer than 7 days after administration. Exclusion criteria were: (1) missing clinical data; (2) missing ALL risk levels and patient’s weight. According to clinical guidelines and previous literature, the elimination delay of MTX was defined as C_24h_ ≥ 10.0 µmol/L, C_48h_ ≥ 1.0 µmol/L, and C_72h_ ≥ 0.1 µmol/L in this study [2, 8, 9, 13, 15–17].

### Feature selection

We consulted the variables that were influential in previous studies on delayed MTX elimination, as evidenced in Additional Table 1. The variables in this study comprised demographic characteristics, clinical features, combination medications, and laboratory test data. The demographic variables included age, gender, and weight, whereas clinical features encompassed emesis, hydrops, immunological classification, ALL risk level, the dosage of MTX, and cell morphological classification. Combination medications consisted of omeprazole, ofloxacin, levofloxacin, and benzylpenicillin sodium. The laboratory test variables included total bilirubin (TBIL), creatinine (Cr), uric acid (UA), albumin (ALB), alanine aminotransferase (ALT), urine PH-value (PH), pressure-controlled ventilator (PCV), white blood cell (WBC), platelet count (PLT), hemoglobin (HGB), prothrombin time (PT), lactate dehydrogenase (LDH), fibrinogen (FIB), cerebrospinal fluid (CSF) transparency, and Pandy’s test.

### Statistical analysis

The patients were randomly divided into a training set and a test set at a ratio of 7:3 using a random number table. The training set was utilized to select predictors and construct the prediction model, while the test set was used to evaluate the performance of the model. All statistical analyses were conducted in R for Windows (version 3.6.1, https://www.r-project.org/) and SPSS 25.0 (IBM Corporation, Armonk, NY, USA). The random forest algorithm was used to fill in missing values that were less than 30%.

**Fig. 1:**
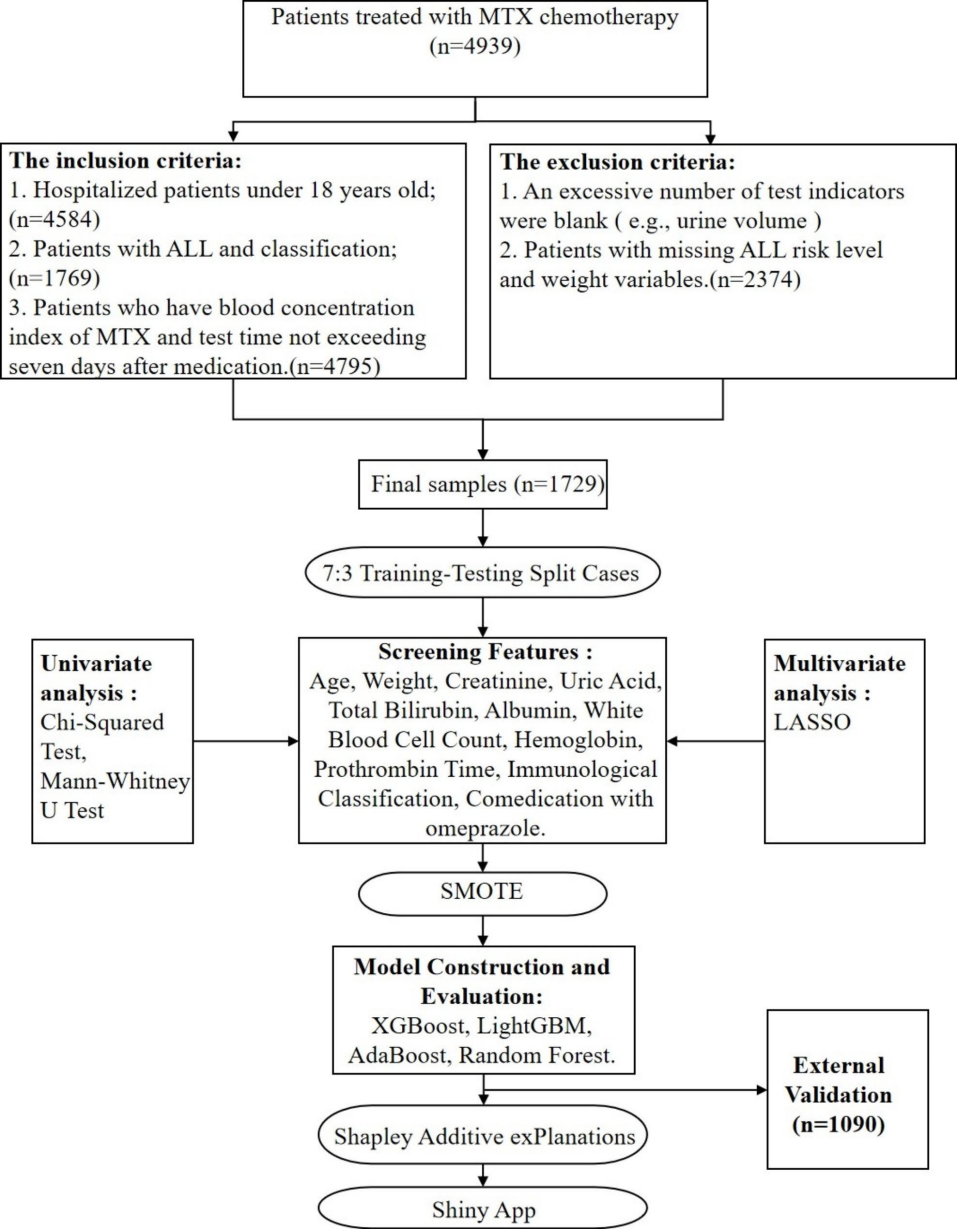
Overall modeling process

Initially, the normality of continuous variables was assessed using the Shapiro-Wilk test. The t-test was utilized for normal data, while the Mann-Whitney test was used for non-normal data in the univariate analysis. Additionally, the Pearson chi-square test was used for categorical variables. The significant indicators selected by univariate analysis were further filtered using the least absolute shrinkage and selection operator (LASSO) regression method. To address the issue of imbalanced data sets, we conducted three different sampling methods on imbalanced datasets. Oversampling, under-sampling and Synthetic Minority Oversampling Technique (SMOTE) was employed to balance the data sets. ML-based prediction models were constructed using the predictors filtered by LASSO. In the model construction, four ML models were developed, including extreme gradient boosting (XGBoost), random forest classifier (RFC), adaptive boosting (AdaBoost), and light gradient boosting machine (LightGBM). The grid search algorithm was employed to determine the optimal parameters of the model. The area under the receiver operating characteristic curve (AUROC) and the area under the precision-recall curve (AUPR) were used to evaluate the model performance. Additionally, SHapley Additive exPlanation (SHAP) was utilized to interpret the chosen model and complete SHAP visualization. The entire statistical analysis process is shown in Fig. 1. In the previous research on the prediction model of MTX delayed elimination, in addition to using ML, logistic regression was also used. So, we also build a logistic regression nomogram to compare its performance with optimal ML. Finally, we use an external validation set to ensure the generalization and consistency of the model.

## Results

### Study population

In our research’s dataset (1729 cases), there were 329 and 1400 cases with and without metabolic delays, respectively. After proportionally dividing the dataset with a ratio of 7:3, the training set (1210 cases) comprised 230 patients with metabolic delay and 980 patients without metabolic delay. In the test set (519 cases), 99 patients experienced metabolic delay and 420 patients did not. The external validation set includes 1090 data cases.

### Feature selection and data preprocessing

**Fig. 2:**
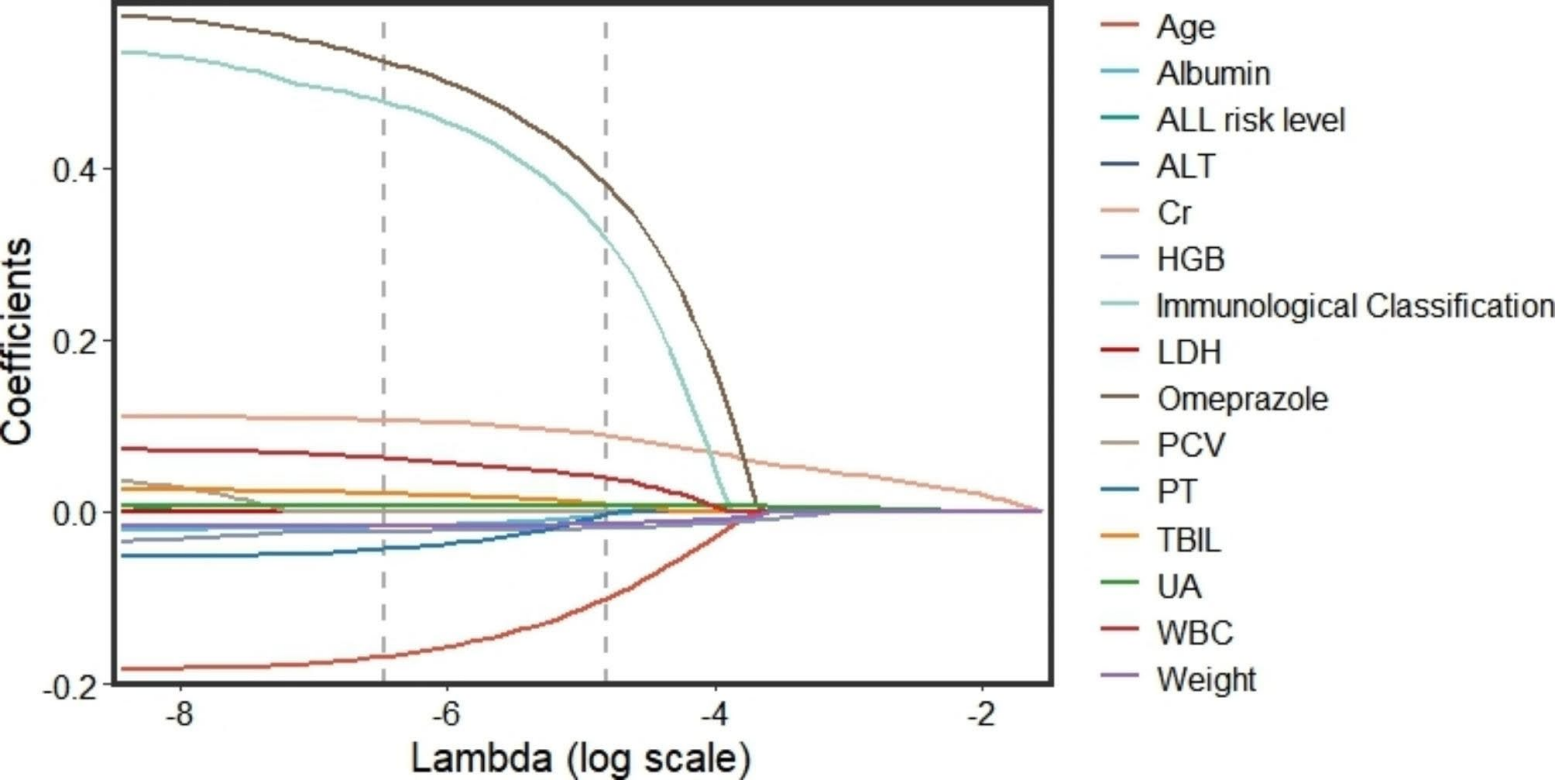
Coefficient regression graph The horizontal coordinate is the magnitude of the λ value in the LASSO regression model. As the λ value changes, the later the coefficient is compressed to zero the more influential the variable is. The graphs show that age, TBIL, and Immunological Classification are highly significant

**Fig. 3:**
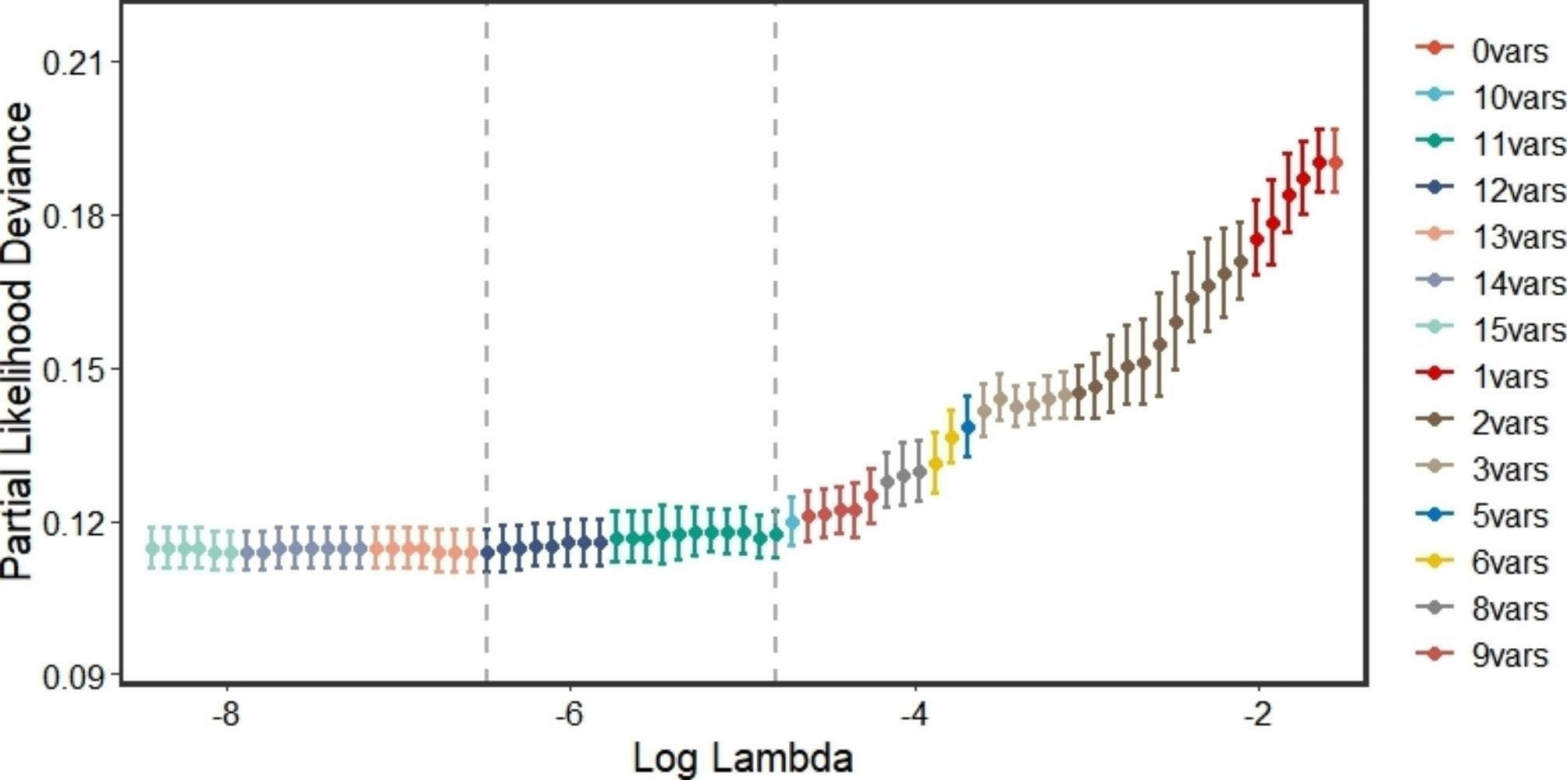
Cross validation curve The dashed lines indicate the particular λ values, Lambda.min and Lambda.1se. The former represents higher accuracy using the corresponding number of features, i.e., a few more features are used; the latter represents the most straightforward model constructed, i.e., fewer features are used

Upon conducting the Shapiro-Wilk test, it was found that all variables were non-normally distributed (as per Additional Table 2). Consequently, we employed the Mann-Whitney U test to compare the continuous variables. Our analysis revealed that age, weight, Cr, UA, TBIL, ALB, ALT, PCV, WBC, HGB, LDH, and PT were statistically significant between the two groups. Furthermore, the Chi-square test indicated that immunological classification, ALL risk level, and co-medication with omeprazole displayed significant differences between the two groups (as illustrated in Table 1). We subsequently performed a LASSO regression analysis on the 15 significant predictors identified through univariate analysis. The paths of the coefficients with different log-transformed λ values in LASSO regression model was displayed in Fig. 2, which clearly demonstrates the significance of several variables, with the influence on delayed MTX elimination increasing as the line moves closer to zero. Moreover, the cross-validation error plot of the LASSO regression model was depicted in Fig. 3. To create a more simplified model, we selected the top 11 variables that had the greatest impact on the outcome. Ultimately, the LASSO method identified eleven indicators, including age, weight, Cr, UA, TBIL, ALB, WBC, HGB, PT, immunological classification, and co-medication with omeprazole, which were used to develop our predictive models.

**Table 1:** Characteristics of patients with and without delayed MTX elimination

Indictors	All Cohorts (n = 1729)		Training Cohorts (n = 1210)		p
	Without metabolic delay (n = 1400)	With metabolic delay (n = 329)	Without metabolic delay (n = 980)	With metabolic delay (n = 230)	
**Baseline information**					
Age (year)	9.0 (8.0–13.0)	11.0 (9.0–14.0)	9.0 (8.0–13.0)	11.0 (9.0-14.3)	**< 0.001**
Weight (kg)	17.5 (14.0–25.0)	22.0 (16.0–30.0)	17.0 (14.0-25.5)	22.5 (16.0–31.0)	**< 0.001**
Sex					
Male	810 (57.86)	200 (60.79)	562 (57.35)	138 (60.00)	0.510
Female	590 (42.12)	129 (39.21)	418 (42.65)	92 (40.00)	
**Clinical features**					
Emesis	22 (1.57)	2 (0.61)	964 (98.37)	230 (100.00)	0.103
Hydrops	41 (2.93)	7 (2.13)	948 (96.73)	226 (98.26)	0.312
Cell Morphological Classification					
L1	518 (0.37)	118 (35.87)	362 (36.94)	89 (38.70)	0.048
L2	788 (56.29)	202 (61.40)	550 (56.12)	135 (58.70)	
L3	94 (6.71)	9 (2.74)	68 (6.94)	6 (2.61)	
ALL risk level					
Standard risk	3 (0.21)	0 (0.00)	2 (0.20)	0 (0.00)	**< 0.001**
Low risk	836 (59.71)	122 (37.08)	591 (60.31)	88 (38.26)	
Intermediate risk	523 (37.36)	205 (62.31)	363 (37.04)	140 (60.87)	
High risk	38 (2.71)	2 (0.61)	24 (2.45)	2 (0.87)	
Immunological Classification					
B-ALL	1306 (93.29)	280 (85.11)	914 (93.27)	198 (86.09)	**< 0.001**
T-ALL	94 (6.71)	49 (0.15)	66 (6.73)	32 (13.91)	
Dose (g/m²)	3.02 (2.96–4.04)	3.06 (2.90–4.96)	3.02 (2.95-4.00)	3.03 (2.90–4.95)	0.056
**Laboratory test**					
TBIL (µmol/L)	9.3 (6.7–12.5)	10.7 (7.7–14.7)	9.5 (6.7–12.6)	10.5 (7.6–14.7)	**< 0.001**
Cr (µmol/L)	27.0 (22.0–34.0)	49.0 (37.0-65.5)	27.0 (22.0–34.0)	48.0 (37.0-66.3)	**< 0.001**
UA (µmol/L)	222.0 (187.3–261.0)	300.0 (245.0-354.5)	222.0 (189.0-261.0)	299.5 (241.5–351.0)	**< 0.001**
ALB (g/L)	45.2 (42.7–47.2)	43.8 (41.0-46.4)	45.2 (42.4–47.2)	43.8 (41.4–46.4)	**< 0.001**
ALT (IU/L)	20.9 (14.0-40.15)	17.2 (28.2–58.2)	20.95 (13.9–38.7)	25.9 (16.2–55.0)	**< 0.001**
PH	6.5 (6.0–7.0)	6.5 (6.0–7.0)	6.5 (6.0–7.0)	6.5 (6.0–7.0)	0.035
PCV (%)	32.6 (30.1–34.7)	30.8 (27.9–33.2)	32.5 (29.9–34.6)	31.1 (28.1–33.4)	**< 0.001**
WBC (10^9^/L)	3.30 (2.40–4.55)	4.12 (2.67–5.80)	3.28 (2.40–4.52)	4.21 (2.57–6.13)	**< 0.001**
PLT (10^9^/L)	295.0 (224.0-383.0)	212.0 (278.0-359.0)	293.0 (223.0-382.8)	272.5 (207.8-354.8)	0.016
HGB (g/L)	107.0 (98.0-114.0)	102.0 (91.0-111.0)	108.0 (99.0-114.0)	102.0 (91.8–112.0)	**< 0.001**
PT (second)	11.74 (11.20–12.20)	11.6 (10.8–12.0)	11.79 (11.20–12.20)	11.6 (10.8–12.0)	**< 0.001**
LDH (IU/L)	247.3 (216.7–282.0)	259.0 (231.3-291.9)	247.3 (216.3-283.3)	256.6 (228.7-289.7)	**< 0.001**
FIB (g/L)	2.29 (1.97–2.70)	2.37 (2.07–2.80)	2.29 (1.97–2.70)	2.37 (2.09–2.80)	0.001
CSF transparency					
Clear	1342 (95.86)	321 (97.57)	936 (95.51)	223 (96.96)	0.543
Micro-turbidity	38 (2.71)	3 (0.91)	27 (2.76)	3 (1.30)	
Turbidity	18 (1.86)	5 (1.52)	15 (1.53)	4 (1.74)	
Turbid with clots	2 (0.14)	0 (0.00)	2 (0.20)	0 (0.00)	
Pandy’s test					
(-)	1277 (91.21)	305 (92.71)	888 (90.61)	213 (92.61)	0.239
(±)	58 (4.14)	6 (1.82)	42 (4.29)	4 (1.74)	
(+)	28 (2.0)	13 (3.95)	22 (2.24)	9 (0.39)	
(++)	28 (2.0)	5 (1.52)	23 (2.35)	4 (1.74)	
(+++)	7 (0.50)	0 (0.00)	3 (0.31)	0 (0.00)	
(++++)	2 (0.14)	0 (0.01)	2 (0.20)	0 (0.00)	
Cl (mg/L)	122.1 (119.9-124.1)	122.2 (120.3-124.6)	122.0 (119.8-124.1)	122.2 (120.3–124.0)	0.454
**Drug combination**					
Omeprazole	48 (3.43)	57 (17.33)	28 (2.86)	36 (15.65)	**< 0.001**
Ofloxacin	13 (0.93)	0 (0.00)	12 (1.22)	0 (0.00)	0.189
Benzylpenicillin sodium	7 (0.50)	2 (0.61)	4 (0.41)	2 (0.87)	0.708
Levofloxacin	13 (0.93)	0 (0.00)	12 (1.22)	0 (0.00)	0.189

Abbreviations: (-): negative, transparent, (±): weakly positive, between transparent and gonorrhea, (+): mild gonorrhea, (++): moderate gonorrhea, (+++): intense gonorrhea, (+++): milky gonorrhea

### Model evaluation and interpretation

The variables selected previously were utilized as input variables to establish a prediction model for delayed MTX elimination, with the occurrence of delayed MTX elimination being designated as the outcome event (yes = 1, no = 0). Ultimately, a total of 230 patients with delayed MTX elimination and 980 patients without delayed MTX elimination were included in the training set to develop the predictive model. The test set was then used to validate the predictive ability of the established model. The performance of the delayed MTX elimination risk prediction models with different sampling methods are showed in Additional Table 3.

**Fig. 4:**
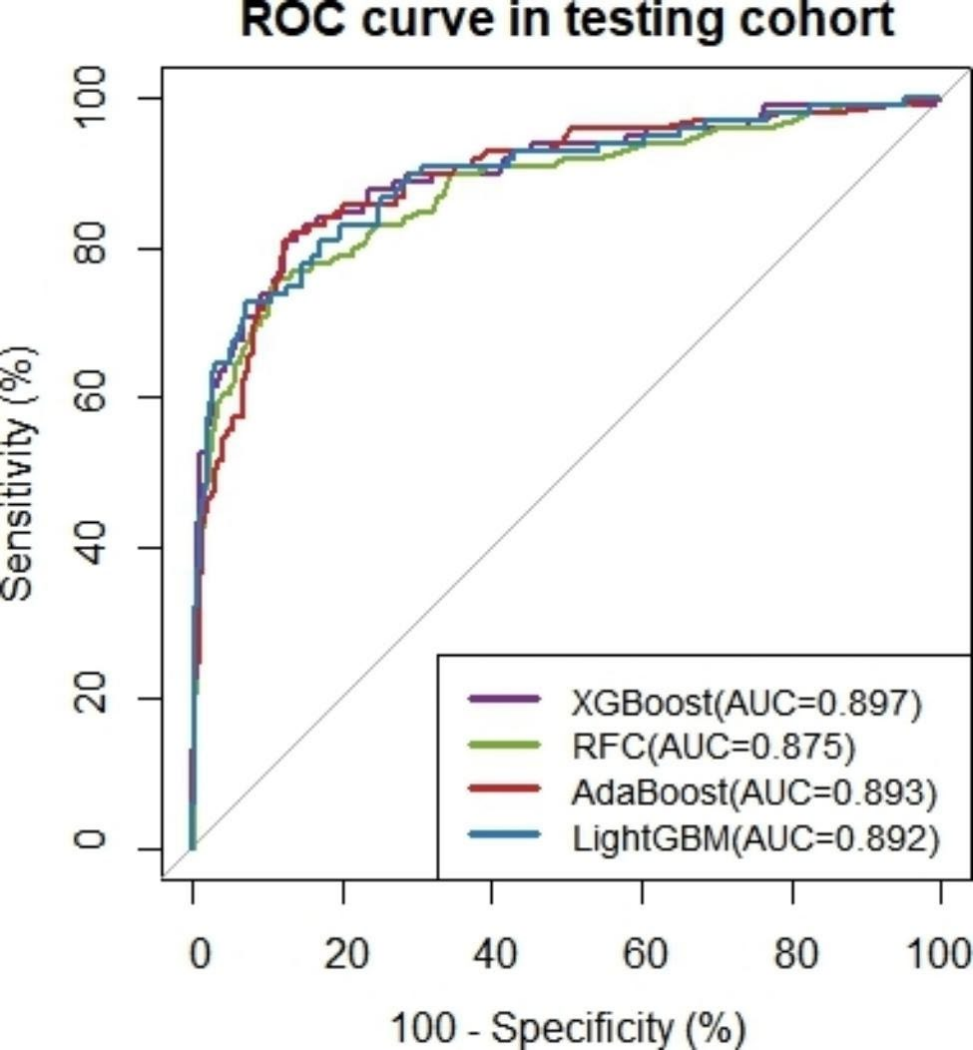
ROC curve of 4 ML models for predicting MTX delayed elimination in the testing set

**Fig. 5:**
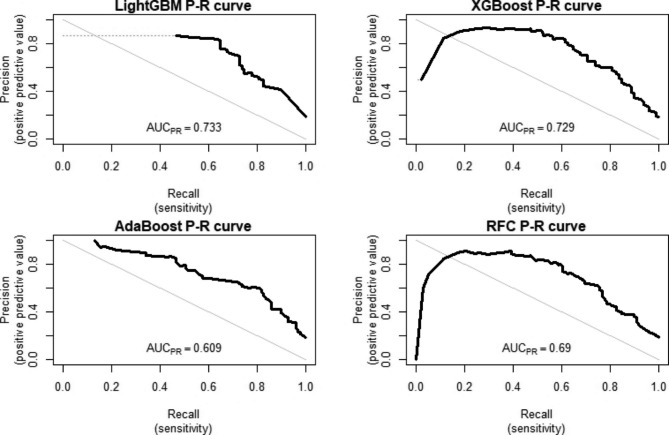
PR curve of 4 ML models for predicting MTX delayed elimination in the testing set

We chose the XGBoost model sampled by SMOTE as the optimal model for this study. The AUROC performance of the delayed MTX elimination risk prediction model with SMOTE is illustrated in Fig. 4. The AUPR value is more sensitive to sample distribution, and the precision-recall (P-R) curve to showcase the model’s precision and recall performance (Fig. 5). The AUROC value of XGBoost using SMOTE is 0.897(0.857–0.937) and it had an area under the P-R curve (AUPR) of 0.729. In addition, XGBoost sensitivity in SMOTE is 0.808. The higher the sensitivity, the better the model’s ability to correctly identify delayed elimination, and the lower the missed diagnosis rate. The comparison process for selecting the optimal model can be found in Additional File 1. We apply the optimal model to predict external validation sets. It was found that AUROC = 0.788 (0.753–0.822) in external validation, indicating good discrimination ability. We apply the optimal model to predict external validation sets. We used the optimal model to predict the external validation set, and the model demonstrated good performance. Among them, AUROC = 0.788 (0.753–0.822), AUPR = 0.648, specificity = 0.813 (0.780–0.840), sensitivity = 0.680 (0.625–0.735).

**Fig. 6:**
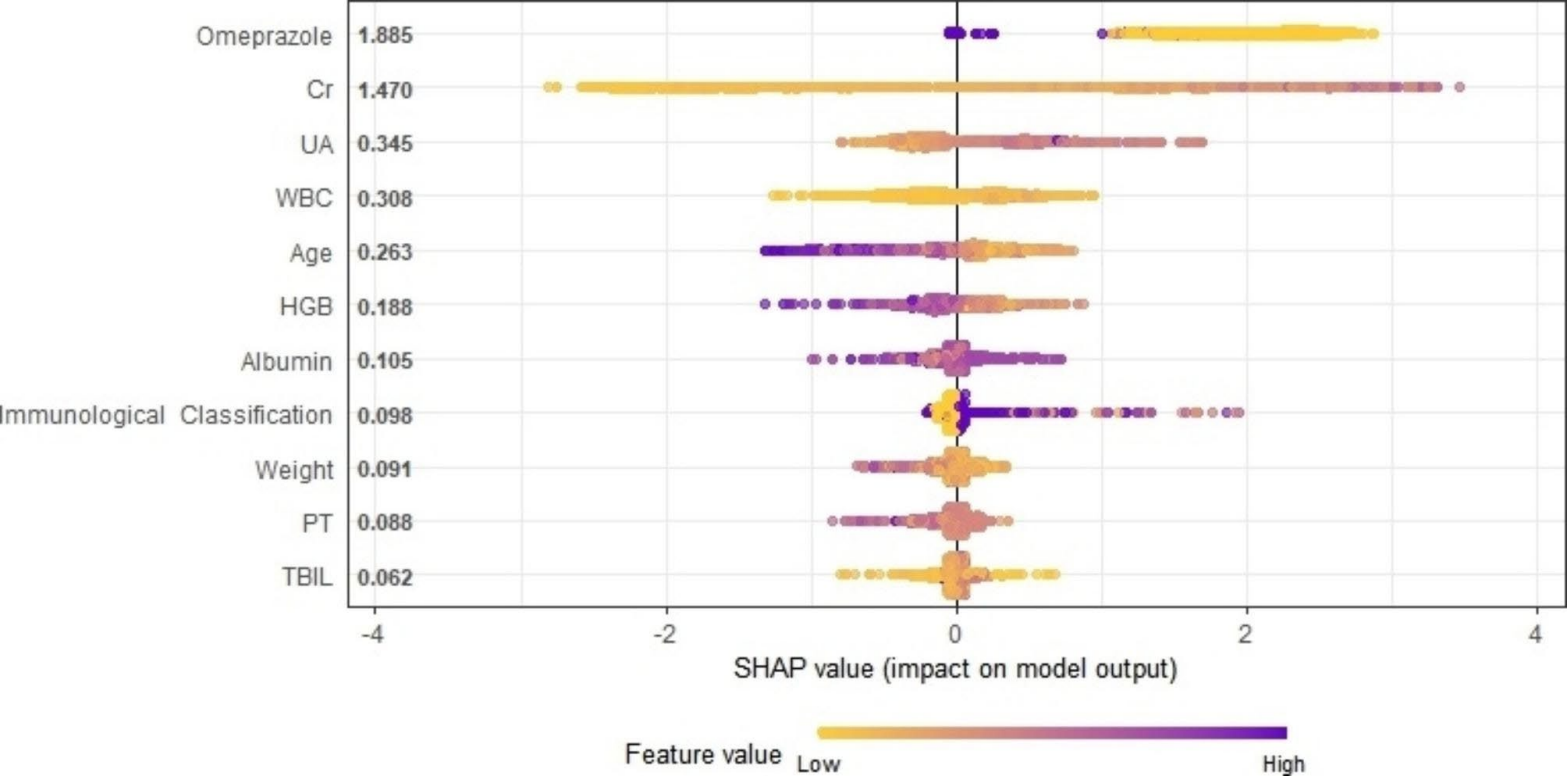
Global Shapley Additive Explanations (SHAP) interpretation for XGBoost The influence distribution of features on model output. The vertical axis is sorted according to the sum of SHAP values of all samples, and the horizontal axis is SHAP value. Each point represents a sample

As illustrated in Fig. 6, the summary graph of SHAP elucidates the prediction of all samples. The SHAP values of each sample’s variable were plotted by scatter plot, and the relationship between SHAP values and outcomes was analyzed. In the XGBoost model, the SHAP summary plot ranked the importance of delayed MTX elimination variables as co-medication with omeprazole, Cr, UA, WBC, HGB, Age, HGB, ALB, immunological classification, weight, PT and TBIL. Additionally, a dependence plot was generated to assess the relationship between the variables and the predicted influence (Additional Figs. 1–11). The dependency graph lucidly portrays how individual variables affect the model’s predictions.

**Fig. 7:**
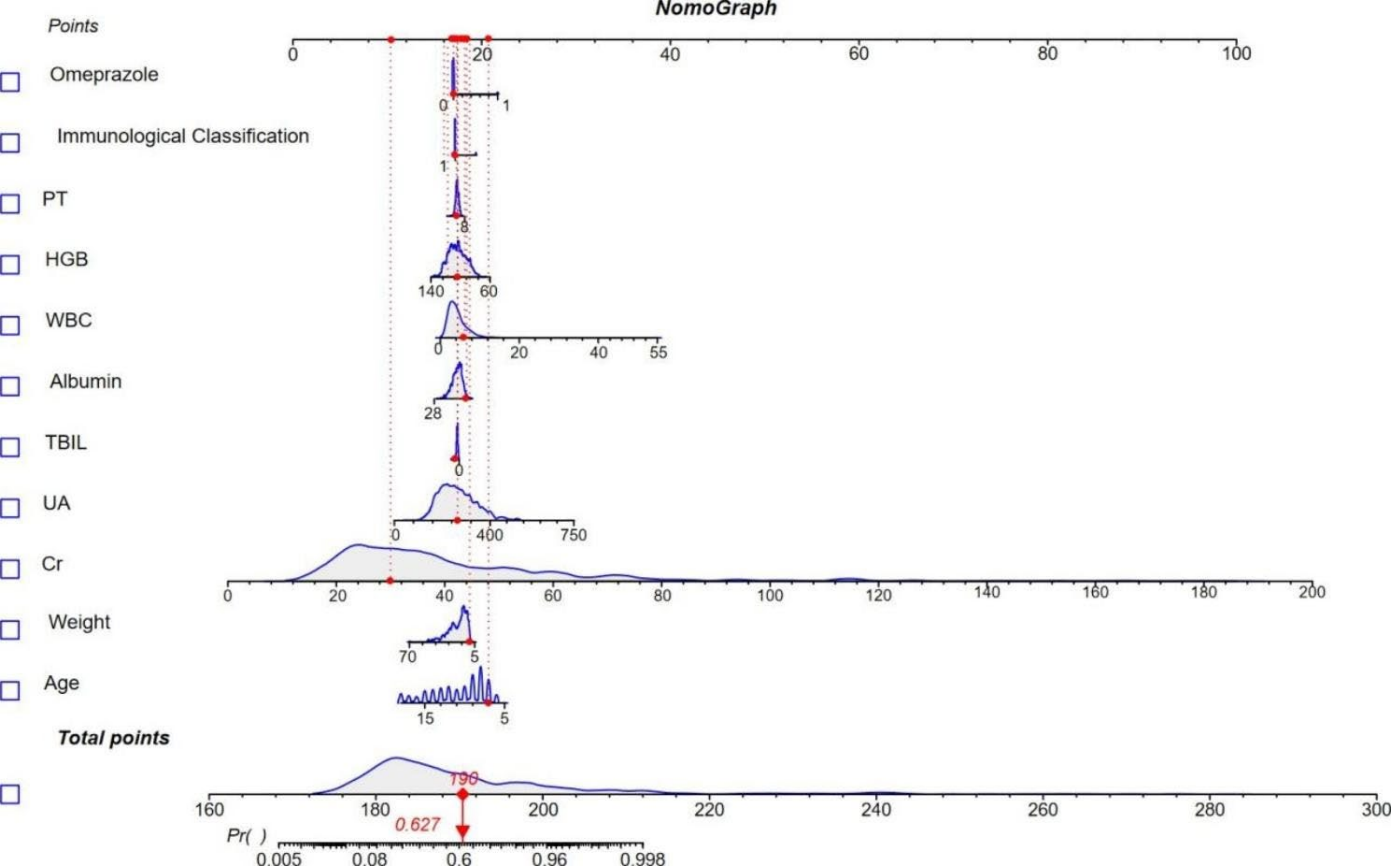
A constructed nomogram for prediction of delayed MTX elimination in Pediatric ALL Patients

We constructed a Logistic regression nomogram using the 11 screened indicators. Figure 7 shows an example of using nomogram to predict MTX delayed elimination. The total score corresponds to the probability value on the risk axis, and a higher total score indicates a higher risk of MTX delayed elimination. We evaluated the nomogram with an AUROC of 0.886(0.844–0.929) as shown in Additional Fig. 12.

## Discussion

Several research studies have illustrated that prolonged elimination after administering HD-MTX to children with ALL may result in serious adverse effects, particularly in those with atypical renal function [2, 5–8, 10]. We formulated a risk assessment algorithm for predicting delayed MTX elimination based on pre-medication information. This can facilitate healthcare professionals in recognizing the possibility of delayed MTX elimination in children with ALL.

In this study, age, weight, Cr, UA, TBIL, ALB, WBC, HGB, PT, immunological classification, and concurrent use of omeprazole were recognized as risk factors for delayed MTX elimination. Most of these autonomous risk factors have been reported in preceding research [8, 13–15, 17–25]. For instance, Nakano T discovered that age, MTX dosage, and TBIL were independent risk factors for delayed MTX elimination [8]. Xu’s research revealed that scrutinizing serum Cr concentration can proficiently anticipate the delay of MTX elimination, and that patients with delayed metabolism have elevated serum Cr levels [22]. A Japanese study found that serum UA levels were correlated with nephrotoxicity prompted by delayed MTX elimination [23]. Another analysis indicated that MTX toxicity could be engendered by combining proton pump inhibitors (such as omeprazole), penicillin family antibiotics, and specific antimicrobial agents [24–27]. We have retained most of the previous studies on influencing factors, while additionally incorporating FIB, PT, chloride in cerebrospinal fluid, and cerebrospinal fluid transparency. These parameters are easily obtainable in medical facilities, and the multifarious possibilities of causing MTX metabolism delay are exhaustively contemplated. For instance, HD-MTX therapy will prolong thrombin time and diminish FIB [28]. Additionally, distinct dosages of MTX exhibit notable drug concentrations in serum and cerebrospinal fluid [29]. The predictors WBC, HGB, and PT are seldom mentioned in preceding studies and require further validation.

Recently, ML techniques have garnered increasing attention in clinical research and emerged as a powerful instrument for addressing numerous healthcare problems [30–32]. In this investigation, we compared the performance of different ML models in different sampling methods for imbalanced data. Among these models’ evaluation, we found that the XGBoost in SMOTE and LightGBM in oversampling were comparable in performance. However, XGBoost demonstrated the better AUPR value and sensitivity. Nitesh Chawla et al. described that smote works by selecting the nearest instances in the feature space, drawing a line between the instances in the feature space, and drawing a new sample along a point of the line [33]. Consequently, we ultimately opted for XGBoost in SMOTE to construct the final prediction model. XGBoost is extensively utilized by data scientists and delivers the most cutting-edge outcomes on a plethora of issues. For instance, XGBoost forestalls overfitting and has the ability to handle voluminous data [34]. Luu Ho Thanh Lam et al. selected XGBoost as the optimal model after SMOTE, to classify the molecular subtypes of low-grade glioma [35]. Nwanosike EM et al. evaluated the advancements of ML algorithms in clinical applications, and the XGBoost algorithm exhibited the highest potential for clinical implementation [36]. We have also implemented the optimal prediction model on the web page to provide a reliable tool for clinical medical professionals and researchers. The web page address is https://cqmugj.shinyapps.io/mtx_jc/.

We constructed a nomogram, which was commonly used in previous studies to predict MTX delayed elimination. We found that the AUROC value using the nomogram was smaller than that of the optimal model (XGBoost). On the other hand, nomogram is a non-parametric model that requires the total score to obtain the probability. And it can’t automatically calculate the result, which is a bit inconvenient compared to ML. In addition, the model’s AUROC and specificity after external validation indicated that it had good discrimination and a low misdiagnosis rate. And the result also reflected the transportability and generalization ability of the model. On the other hand, it indicates that the model has good consistency in different time periods compared to the model development queue.

The current research is mainly to accurately diagnose the adverse reaction or MTX delayed elimination by using the post medication test index of methotrexate combined with ML. We summarize some similar studies and draw a Table 2. For example, Hu et al. created an ML-based model for predicting low-dose MTX-related hepatotoxicity with an AUC of 0.97 but only accuracy of 0.64 [37]. Zhan et al. employed an artificial intelligence algorithm to forecast neutropenia and fever caused by high-dose MTX in children with B-cell ALL, with an AUC of 0.870 [38]. The performance of our model is similar to that of Zhan M et al. [7], but inferior to Schmidt, D [13]. In addition, we summarized some researches on the analysis of MTX delayed elimination factors in recent years (see Additional Table 1). However, few studies have integrated the identified risk factors and applied them directly to the prediction of delayed MTX elimination. Zhan M et al. used hematocrit, risk classification, dose, SLC19A1 rs2838958, and sex indicators to develop a prediction model for delayed elimination of MTX. The highest AUC of the model was 0.807 (95% CI, 0.724–0.889) [7]. They used fewer variables and included genetic factors to build a prediction model with better performance. However, our predictors are easily obtainable and it is of great value in identifying MTX metabolic delay.

Nonetheless, the study has certain limitations. Firstly, the incidence, treatment, and individual differences in ALL across different regions may hinder the applicability of the model. Secondly, some variables, such as the genetic characteristics of the affected children and their living environment, have not been included. Thirdly, our study was retrospective research, the examination of some cases was done with inadequate equipment and training, and some indicators with missing values greater than 30% (e.g. urine volume) were not included in the model. Finally, the generalization ability of the model should be further confirmed through multi-center external validation in future studies.

**Table 2:** Summary table of machine learning applied to MTX delayed elimination or Adverse reactions

Title	Authors	Screened Feature	Sample size	Algorithms	AUROC
Risk prediction for delayed clearance of high-dose methotrexate in pediatric hematological malignancies by machine learning [7]	Zhan M	(1) Hematocrit, risk classification, dose, SLC19A1 rs2838958, sex, dose (2) SLC19A1 rs2838958, dose, sex	205	(1) C5.0 decision tree + SMOTE; (2) Nomogram	(1) AUROC = 0.807 (95% CI 0.724–0.889) (2) AUROC = 0.690 (95% CI 0.594–0.787)
Predictive analysis of methotrexate elimination delay based on logistic regression model and ROC curve [13]	Wang Yang	SLCO1B1 T521C	82	Logistic regression	AUROC = 0.751 (0.627–0.875)
Plasma creatinine as predictor of delayed elimination of high-dose methotrexate in childhood acute lymphoblastic leukemia: A Danish population-based study [15]	Schmidt, D	(1) Absolute increase in 36 h Plasma Creatinine (2) Relative increasein 36 h Plasma Creatinine (3) Infusion plasma MTX concentration	218	Linear regression	(1) AUROC = 0.930 (95%CI 0.910-0-960) (2) AUROC = 0.930 (95%CI 0.910-0-960) (3) AUROC = 0.810 (95%CI 0.750-0-860)
Risk factors for high-dose methotrexate-induced nephrotoxicity [22]	Shinichiro Kawaguchi	Urine pH at day 1	88	Logistic regression	0.750 (95% CI 0.573–0.927)
Predicting Hepatotoxicity Associated with Low-Dose Methotrexate Using Machine Learning [37]	Hu, Qiaozhi	BMI, age, number of drugs and comorbidities, doses of folic acid, antibiotic use, gender, immunosuppressive agents, Glucocorticoid use, First MTX use, Drinking, Type 2 diabetes, Chinese traditional medicine, Dose of folic acid, Infectious liver disease, history of kidney disease	782	(1) XGBoost (2) AdaBoost (3) CatBoost (4) GBDT (5) LightGBM (6) TPOT (7) RF (8) ANN	(1)0.94 (2)0.69 (3)0.91 (4)0.53 (5)0.87 (6)0.78 (7)0.97 (8)0.65

Abbreviations: SMOTE, Synthetic Minority Over-Sampling Technique; RF: Random Forest; XGBoost: eXtreme gradient boosting; AdaBoost: Adaptive boosting; LightGBM: Light gradient boosting machine; AUROC: Area under the receiver operating characteristic curve; CatBoost, Categorical boosting; GBDT, Gradient Boosting Decision Tree; TPOT, Tree-based Pipeline Optimization Tool; ANN, Artificial Neural Network; BMI, Body Mass Index

## Conclusions

In summary, this investigation illustrates that factor such as age, body weight, creatinine, uric acid, total bilirubin, albumin, white blood cell count, hemoglobin, prothrombin time, cellular morphological classification, and concomitant use of omeprazole could be served as predictors for delayed MTX elimination. Through the application of XGBoost after SMOTE, delayed MTX elimination can be effectively identified in children diagnosed with ALL. Our predictive model provides a reliable means for monitoring the metabolic delay of MTX, even in the absence of MTX plasma concentration monitoring. By utilizing this tool, medical professionals can take timely targeted measures to prevent the occurrence of MTX-related adverse drug events.

### Electronic supplementary material

Below is the link to the electronic supplementary material.

Supplementary Material 1

Supplementary Material 2

## Data Availability

The data underlying this article will be shared on reasonable request to the corresponding author.

## List of Abbreviations

MTX: Methotrexate
HD-MTX: High-dose methotrexate
ALL: Acute lymphoblastic leukemia
CNS: Central nervous system
NHL: Non-Hodgkin’s lymphoma
AKI: Acute kidney injury
ML: Machine learning
LASSO: Least absolute shrinkage and selection operator
RFC: Random forest classifier
XGBoost: eXtreme gradient boosting
AdaBoost: Adaptive boosting
LightGBM: Light gradient boosting machine
CatBoost: Categorical boosting
GBDT: Gradient Boosting Decision Tree
TPOT: Tree-based Pipeline Optimization Tool
ANN: Artificial Neural Network
BMI: Body Mass Index
SMOTE: Synthetic Minority Oversampling Technique
AUROC: Area under the receiver operating characteristic curve
AUPR: Area under PR curve
SHAP: Shapley Additive exPlanations
Cr: Creatinine
UA: Uric acid
TBIL: Total bilirubin
ALB: Albumin
WBC: White blood cell count
HGB: Hemoglobin
PT: Prothrombin time
ALT: Alanine aminotransferase
PH: Urine PH-value
PCV: Pressure controlled ventilator
PLT: Platelet count
LDH: Lactate dehydrogenase
FIB: Fibrinogen
Cl: Chloride ion
