# Supplementary material for: Predicting delayed methotrexate elimination in pediatric acute lymphoblastic leukemia patients: an innovative web-based machine learning tool developed through a multicenter, retrospective analysis
Source: BMC Med Inform Decis Mak. 2023 Aug 3;23:148. doi: 10.1186/s12911-023-02248-7 (PMC10398990; doi:10.1186/s12911-023-02248-7)
Supplement: Supplementary file 2 — Supplementary Material 2 [file 12911_2023_2248_MOESM2_ESM.docx]

## Additional File 1

**Additional Table 1** Summary table showing researches of delayed MTX elimination or adverse drug events related indicators

| Title | Authors | Screened Feature Summary | Sample size | Tools |
| --- | --- | --- | --- | --- |
| Risk prediction for delayed clearance of high-dose methotrexate in pediatric hematological malignancies by machine learning [7] | Zhan M | Hematocrit, risk classification, dose, SLC19A1 rs2838958, sex, dose | 205 | univariable analysis and, separately, multivariate logistic regression |
| Risk factors for delayed elimination of high-dose methotrexate in childhood acute lymphoblastic leukemia and lymphoma [14] | Nakano, T | First HD-MTX course, lower urine volume per body surface area on the day and the next day of starting HD-MTX, high dose of HD-MTX, higher total bilirubin, and lower expressed as estimated glomerular fltration rate | 269 | Multivariate analysis |
| Plasma creatinine as predictor of delayed elimination of high-dose methotrexate in childhood acute lymphoblastic leukemia: A Danish population-based study [15] | Schmidt, D | Plasma creatinine | 218 | Receiver operating characteristic (ROC) curves |
| Risk factors for high-dose methotrexate associated toxicities in patients with primary central nervous system lymphoma [18] | Sun,K | AKI, ALT, WBC, eGFR, LDH and Co-administration of vindesine | 54 | Chi-square tests, Multivariate logistic regression, Area under the concentration timecurve |
| Low Serum Albumin Level Is a Risk Factor for Delayed Methotrexate Elimination in High-Dose Methotrexate Treatment [19] | Kataoka, T | Low serum albumin | 74 | Logistic analysis |
| Research Progress in Influence Factors of Excretion Delay of High-dose Methotrexate [20] | PANG Lu | Hydrothorax, ascites or postoperative wound effusion, nonsteroidal anti-inflammatory drugs, Antibiotics, Urine pH, vomit | - | Consult the literature, summarize and sort out |
| Serum creatinine and creatinine clearance for predicting plasma methotrexate concentrations after high-dose methotrexate chemotherapy for the treatment for childhood lymphoblastic malignancies [21] | [Xu, WQ](https://www.webofscience.com/wos/alldb/general-summary?queryJson=%5b%7b) | Creatinine clearance (CrCl) and/or serum Cr | 105 | Generalized estimating equations (GEE) |
| Risk factors for high-dose methotrexate-induced nephrotoxicity [22] | Shinichiro Kawaguchi | Low urine pH | 88 | Mann–Whitney U test, Fisher’s exact test and Logistic regression |
| Effects of proton pump inhibitors and famotidine on elimination of plasma methotrexate: Evaluation of drug-drug interactions mediated by organic anion transporter 3 [23] | Katsuya Narumi | PPIs (esomeprazole, lansoprazole, omeprazole and rabeprazole) | 43 | Mann-Whitney U test |
| Predicting Hepatotoxicity Associated with Low-Dose Methotrexate Using Machine Learning [30] | Hu, Qiaozhi | BMI, age, number of drugs and comorbidities, doses of folic acid, antibiotic use, gender, immunosuppressive agents, Glucocorticoid use, First MTX use, Drinking, Type 2 diabetes, Chinese traditional medicine, Dose of folic acid, Infectious liver disease, history of kidney disease | 782 | Mann–Whitney U test and Chi-square test |

**Additional Table 2** S-W Normal distribution test overall description results

|  | Median | Mean | SD | Skewness | Kurtosis | p |
| --- | --- | --- | --- | --- | --- | --- |
| Age | 9 | 10.549 | 3.362 | 0.657 | -0.635 | >0.05 |
| Weight | 18 | 21.207 | 9.899 | 1.544 | 3.224 | >0.05 |
| Dose | 3.018 | 3.464 | 1.368 | 1.459 | 5.481 | >0.05 |
| Cr | 29 | 34.521 | 19.794 | 3.36 | 16.692 | >0.05 |
| UA | 232 | 241.34 | 68.828 | 0.849 | 2.083 | >0.05 |
| TBIL | 9.6 | 10.431 | 5.564 | 1.773 | 6.47 | >0.05 |
| ALB | 45 | 44.592 | 3.632 | -0.457 | 0.3 | >0.05 |
| ALT | 22.7 | 40.555 | 57.262 | 6.619 | 76.495 | >0.05 |
| PH | 6.5 | 6.617 | 0.686 | 0.229 | -0.306 | >0.05 |
| PCV | 32.4 | 31.96 | 3.605 | -0.422 | 0.33 | >0.05 |
| WBC | 3.41 | 4.067 | 2.972 | 6.956 | 98.318 | >0.05 |
| PLT | 291 | 310.175 | 131.918 | 1.078 | 2.305 | >0.05 |
| HGB | 107 | 105.927 | 12.062 | -0.267 | -0.227 | >0.05 |
| LDH | 249.7 | 267.965 | 194.157 | 18.038 | 415.337 | >0.05 |
| PT | 11.7 | 11.693 | 0.853 | 0.225 | 1.146 | >0.05 |
| Cl | 122.1 | 122.127 | 3.311 | 0.315 | 0.945 | >0.05 |
| FIB | 2.29 | 2.416 | 0.633 | 1.56 | 5.399 | >0.05 |

Abbreviations: Cr, creatinine; UA, uric acid; TBIL, total bilirubin; ALB, albumin; ALT, alanine aminotransferase; PH, urine PH-value; PCV, pressure-controlled ventilator; WBC, white blood cell; PLT, platelet count; HGB, hemoglobin; LDH, lactate dehydrogenase; PT, prothrombin time; Cl, Chloride ion; FIB, fibrinogen.

We compared the performance of 4 ML models in different sampled methods in Table 2. XGBoost has the best performance in SMOTE, LightGBM has the best performance in Oversampling, and all evaluation values of under-sampling are relatively low. So, we compare SMOTE and Oversampling optimal models respectively. The AUROC value of XGBoost using SMOTE is 0.897(0.857-0.937), and the AUROC value of LightGBM using oversampling is 0.900. Delong test between the two AUROC was conducted and the p-value was 0.7301 (>0.05). So, there was no significant statistical difference in the AUROC values between them. On the other hand, XGBoost with SMOTE had an area under the P-R curve (AUPR) of 0.729, followed by LightGBM with an AUPR of 0.722. The AUPR value is more sensitive to sample distribution, and the XGBoost AUPR value in SMOTE is higher than the LightGBM in oversampling, resulting in better classifier performance. In addition, XGBoost sensitivity in SMOTE is higher than LightGBM in Oversampling. The higher the sensitivity, the better the model's ability to correctly identify delayed elimination, and the lower the missed diagnosis rate. In summary, we chose the XGBoost model sampled by SMOTE as the optimal model for this study.

**Additional Table 3** Performance evaluation index of different machine learning models with different sampling.

| Sampling method | Evaluating indicator | XGBoost | AdaBoost | LightGBM | RFC |
| --- | --- | --- | --- | --- | --- |
| SMOTE | AUROC | 0.897  (0.857-0.937) | 0.893  (0.854-0.932) | 0.892  (0.851-0.932) | 0.875  (0.831-0.918) |
|  | AUPR | 0.729 | 0.609 | 0.733 | 0.690 |
|  | Sensitivity | 0.808  (0.731-0.886) | 0.808  (0.731-0.886) | 0.727  (0.640-0.815) | 0.758  (0.673-0.842) |
|  | Specificity | 0.874  (0.842-0.906) | 0.879  (0.847-0.910) | 0.929  (0.904-0.953) | 0.883  (0.853-0.914) |
|  | Cut-off | 0.538 | 0.332 | 0.486 | 0.438 |
| Over  Sampling | AUROC | 0.898  (0.860-0.936) | 0.890  (0.848-0.932) | 0.900  (0.866-0.935) | 0.841  (0.789-0.893) |
|  | AUPR | 0.752 | 0.741 | 0.722 | 0.365 |
|  | Sensitivity | 0.838  (0.766-0.911) | 0.828  (0.754-0.903) | 0.788  (0.707-0.868) | 0.758  (0.673-0.842) |
|  | Specificity | 0.826  (0.790-0.862) | 0.848  (0.813-0.882) | 0.902  (0.874-0.931) | 0.869  (0.837-0.901) |
|  | Cut-off | 0.380 | 0.404 | 0.361 | 0.375 |
| Under  Sampling | AUROC | 0.885  (0.846-0.925) | 0.880  (0.837-0.923) | 0.865  (0.817-0.913) | 0.864  (0.819-0.908) |
|  | AUPR | 0.730 | 0.753 | 0.594 | 0.416 |
|  | Sensitivity | 0.798  (0.719-0.877) | 0.768  (0.684-0.851) | 0.747  (0.662-0.833) | 0.758  (0.673-0.908) |
|  | Specificity | 0.824  (0.787-0.860) | 0.890  (0.861-0.920) | 0.917  (0.890-0.943) | 0.864  (0.832-0.897) |
|  | Cut-off | 0.517 | 0.573 | 0.992 | 0.675 |

|  | Dataset partitioning | Data Sampling | Model building |
| --- | --- | --- | --- |
| Seed | 55 | 1234 | 1234 |

**Additional Table 4** Random Seed Table in Different Part


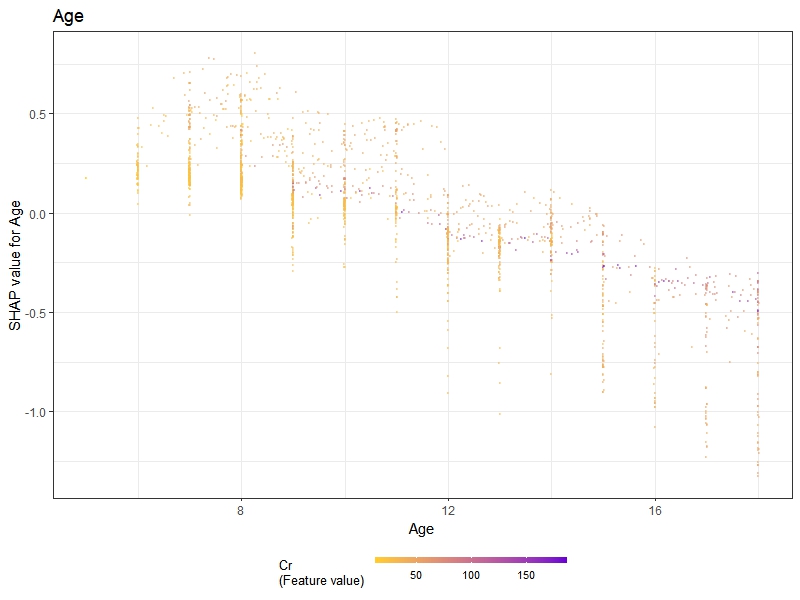


**Additional Fig.1** Dependence plot of Age

**
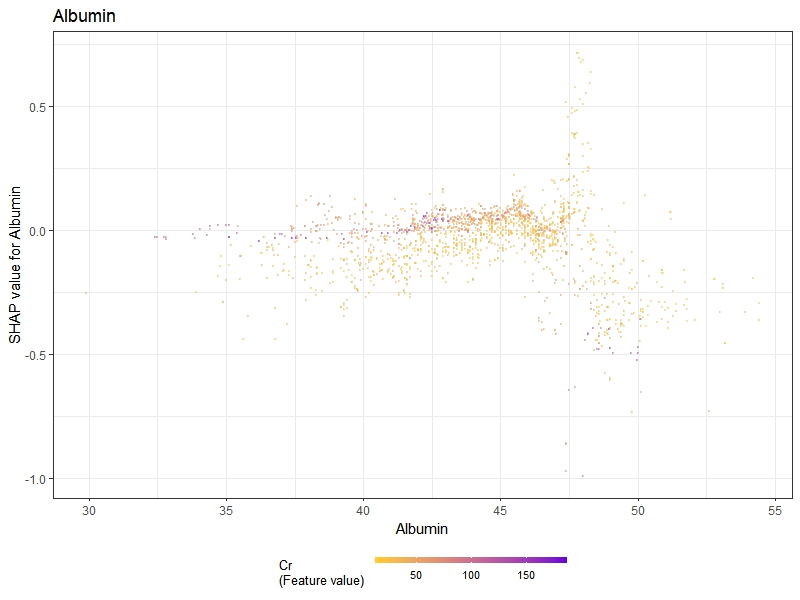
**

**Additional Fig.2** Dependence plot of albumin (ALB)

**
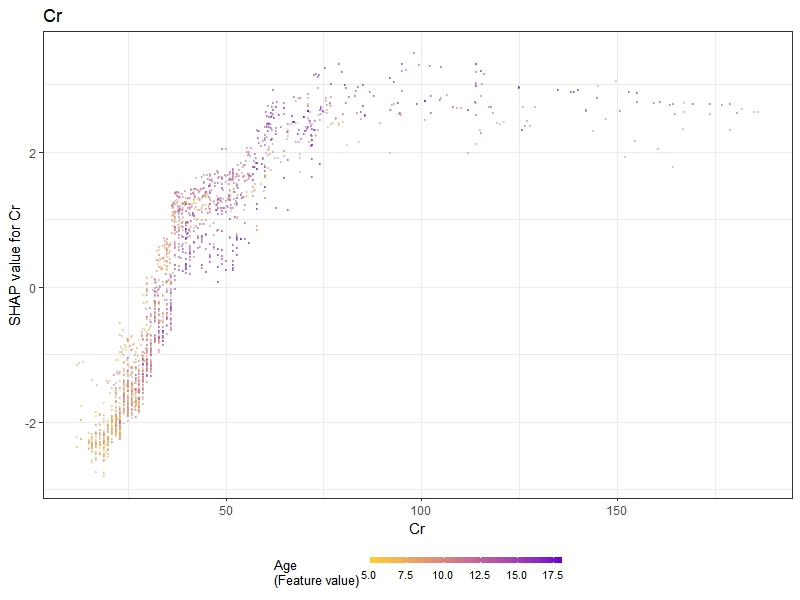
**

**Additional Fig.3** Dependence plot of creatinine (Cr)

**
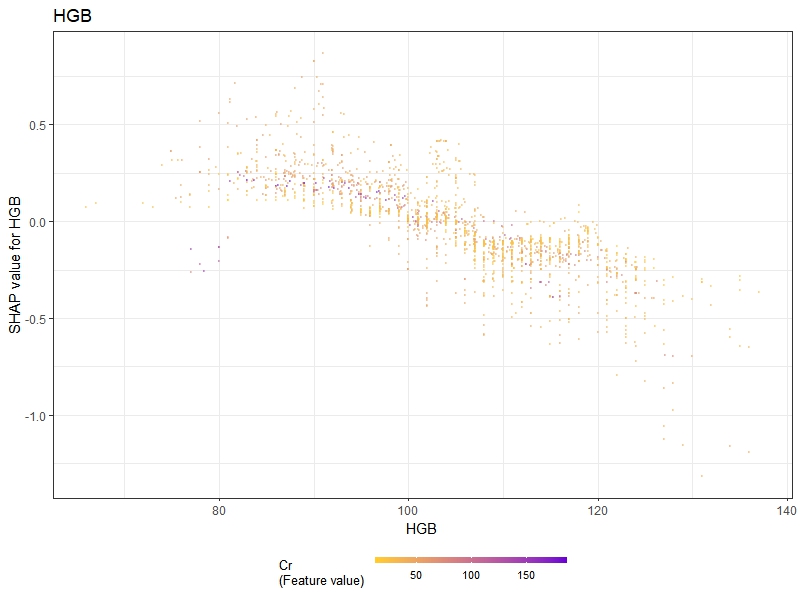
**

**Additional Fig.4** Dependence plot of hemoglobin (HGB)

**
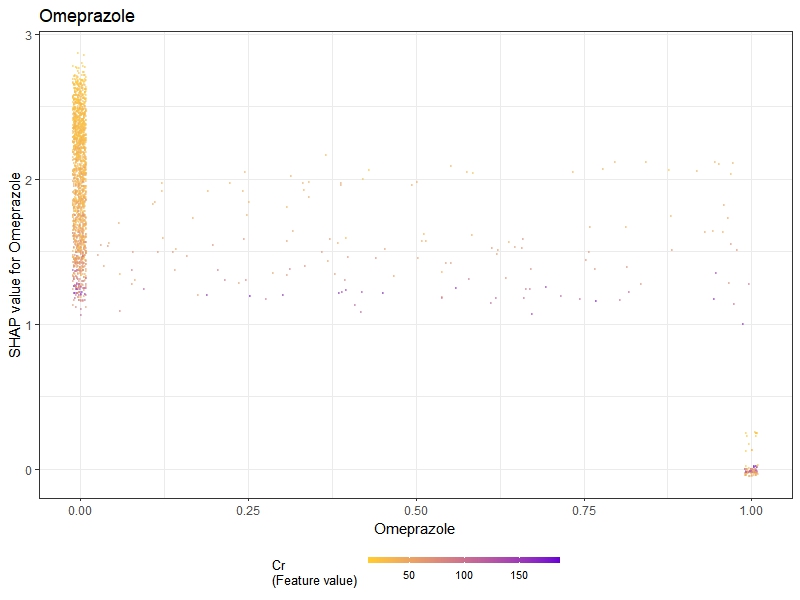
**

**Additional Fig.5** Dependence plot of Omeprazole

**
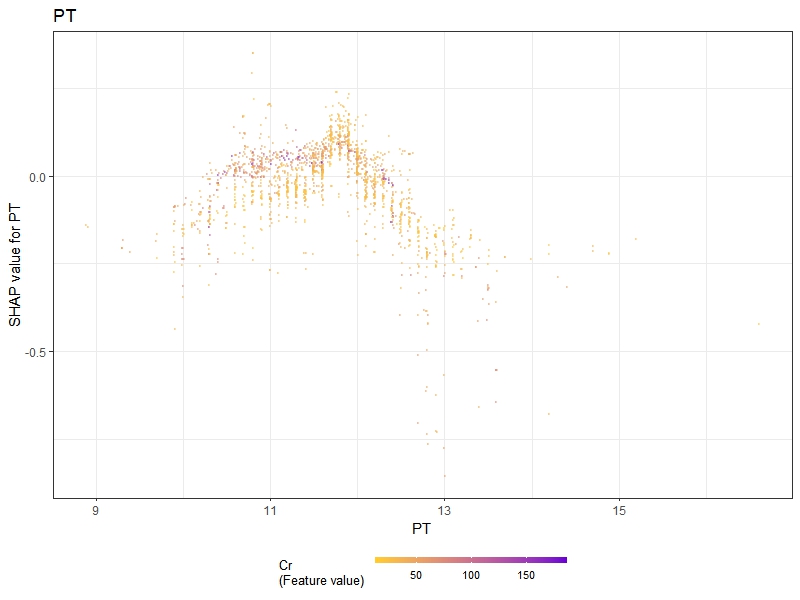
**

**Additional Fig.6** Dependence plot of platelet count (PT)

**
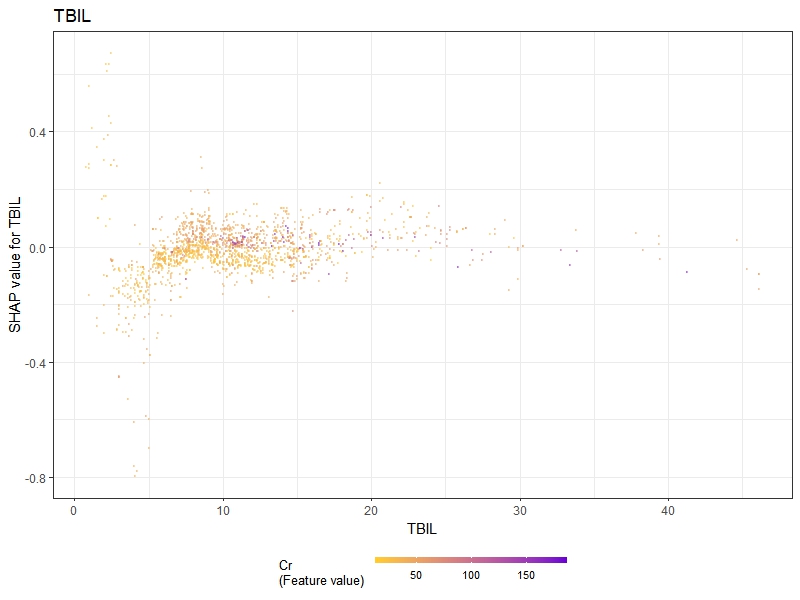
**

**Additional Fig.7** Dependence plot of total bilirubin (TBIL)

**
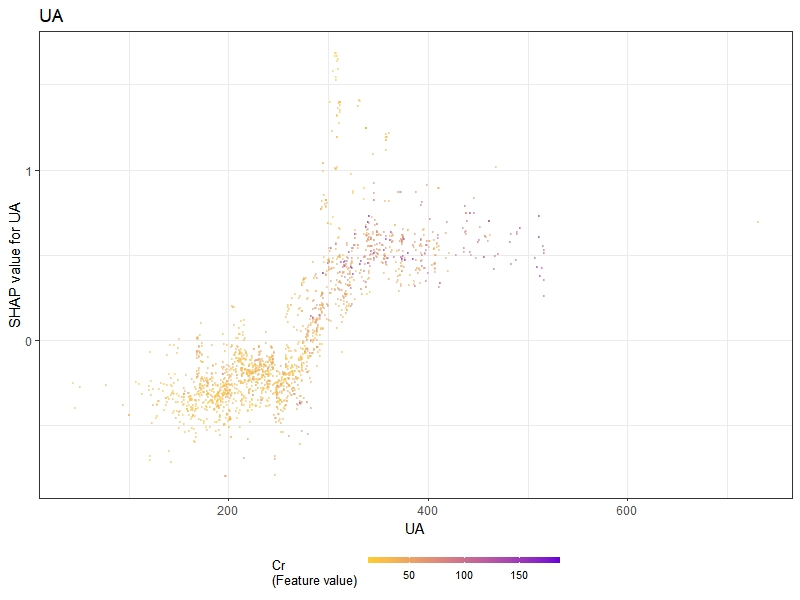
**

**Additional Fig.8** Dependence plot of uric acid (UA)

**
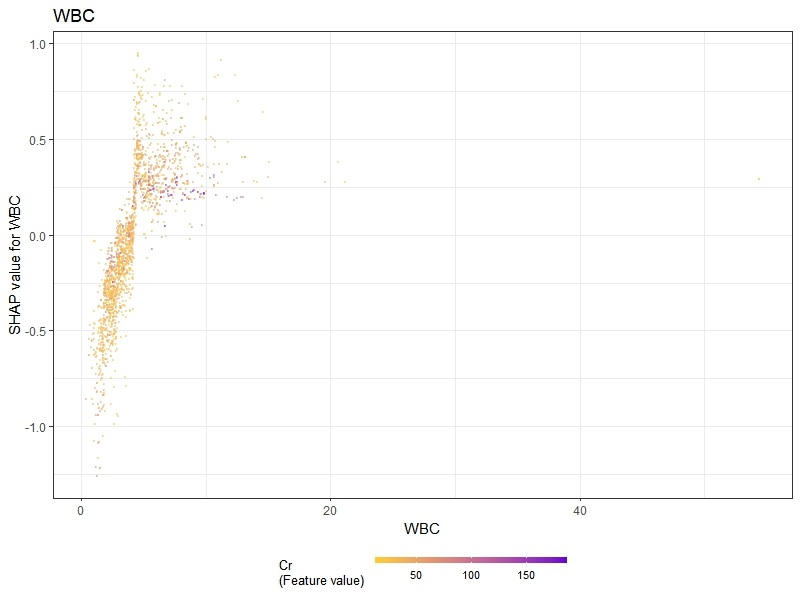
**

**Additional Fig.9** Dependence plot of white blood cell (WBC)

**
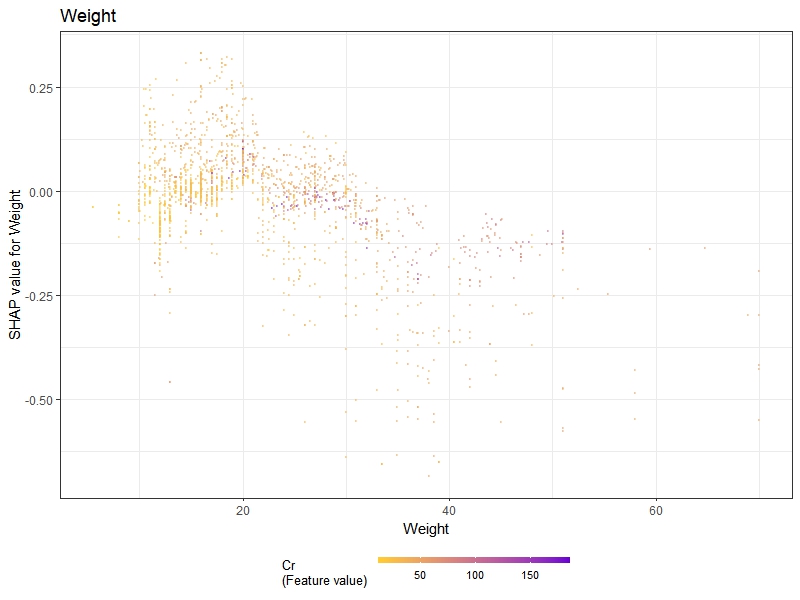
**

**Additional Fig.10** Dependence plot of Weight

**
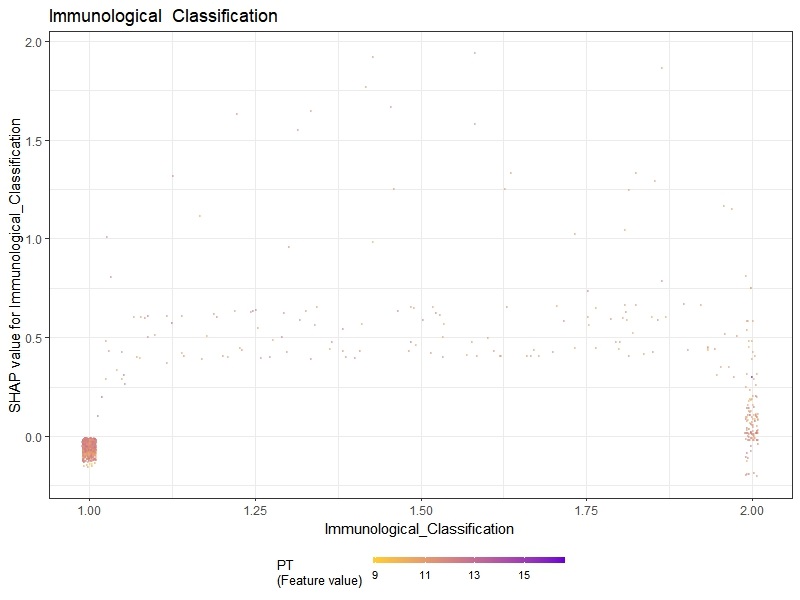
**

**Additional Fig.11** Dependence plot of Immunological Classification


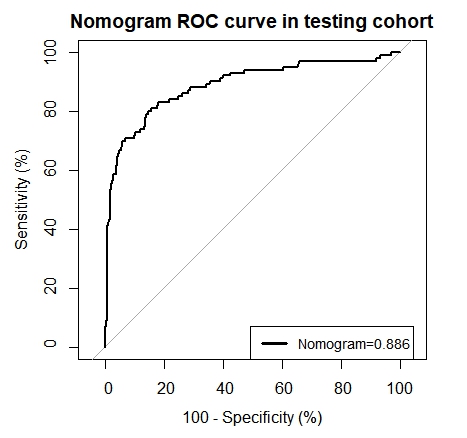


**Additional Fig.12** ROC curve of Nomogram for predicting MTX delayed elimination in the testing set
